# Carotenoids in Fruits of Different Persimmon Cultivars

**DOI:** 10.3390/molecules16010624

**Published:** 2011-01-17

**Authors:** Chunhua Zhou, Daqiu Zhao, Yanle Sheng, Jun Tao, Yong Yang

**Affiliations:** 1 College of Horticulture and Plant Protection, Yangzhou University, Yangzhou 225009, China; Email: chzhou@yzu.edu.cn (C.Z.); 405323488@qq.com (D.Z.); ylsheng2007@163.com (Y.S.); 2 College of Horticulture, Northwest A &F University, Yangling 712100, China; Email: yang.yong521@163.com (Y.Y.)

**Keywords:** carotenoids, provitamin A, HPLC, persimmon

## Abstract

Carotenoids in the peel and the flesh of persimmon fruit were identified, and the contents of carotenoids in the fleshes of 46 different persimmon cultivars were analyzed. The results indicated that 31 specific carotenoids were detected in both cultivars of persimmons, among which nine specific carotenoids were characterized. β-Cryptoxanthin was the most abundant carotenoid among all individual components in both the peel and the flesh, accounting for about 20-30% of the total carotenoids in both cultivars. The contents of total carotenoids in the fleshes of different persimmon cultivars were between 194.61 µg/100g FW and 1,566.30 µg/100g FW. Zeaxanthin was also the most abundant in all persimmon fleshes besides β-Cryptoxanthin, and the total amount of these two components accounted for 37.84-85.11% of the total carotenoids. The RE values in the fleshes of different cultivars also differed greatly. Besides, the stage of maturation was also important factor which could influence the carotenoid content and RE value in the fleshes.

## 1. Introduction

Persimmon (*Diospyros kaki* L.) is an important horticultural crop which has many cultivated varieties. Based on the statistics of FAO (2009), the annual production of persimmon in China is about 2.68 million tons, accounting for about 70.0% of the total world production [1]. Persimmon fruit contains different nutrients and phytochemicals such as carbohydrates, organic acids, vitamins, tannins, polyphenols, dietary fibers and carotenoids etc., which play important roles in the flavor, color, nutritive and pharmaceutical value of the fruit [2,3,4,5].

Carotenoids are biosynthesized by photosynthetic organisms as well as non-photosynthetic bacteria and fungi. The plant carotenoids are dominated by C_40_ isoprenoids with polyene chains containing as many as 15 conjugated double bonds which can be divided into two groups: hydrocarbons (carotenes) and their oxidation derivatives (xanthophylls). These compounds are not only responsible for the yellow, orange and red colors of foods [6], but are also the precursors of vitamin A [7]. Besides, they play multiple roles in the prevention or protection of diseases seriously impairing the human health by oxidative damage, such as heart diseases, cardiovascular diseases, cancers and age-related macular degeneration [8,9].

The persimmon fruit is very captivating, and the color varies in different cultivars from yellow, and orange to deep red. The researchers found that fruit color mainly resulted from the carotenoids, and β-cryptoxanthin was the most abundant among all carotenoids in both the peel and the flesh [3,5]. In the previous studies on carotenoids in persimmon fruit, most of them were based on open-column chromatography or the combination of column chromatography and thin layer chromatography [5,10,11,12,13]. Subsequently, high performance liquid chromatography (HPLC) equipped with ultraviolet/visible light detector and C_18_ reverse phase column was also applied to analyze the carotenoids. However, the cultivars covered were few [14,15], and only a few specific components such as α-carotene, β-carotene and β-cryptoxanthin were analyzed [5,16,17]. Therefore, it is very important to obtain the more detailed information about the carotenoids in persimmon fruit, especially the flesh, the edible part of fruit. The objective of this research was to identify the carotenoids in the peel and the flesh of persimmon fruit and compare the differences between the compositions and contents of carotenoids in the fleshes of different persimmon cultivars using HPLC-PDAD with a C_30_ reverse phase column. Besides, the effect of different stage of maturation on the contents of carotenoids in the flesh of persimmon fruit was also investigated.

## 2. Results and Discussion

### 2.1. Evaluations of fruit quality indexes and color

The fruit size, shape, TSS and titratable acid content varied due to the different cultivars regardless of whether they were non-astringent persimmons or astringent persimmon (Table 1). The difference of the fruit shapes among all the astringent persimmon cultivars was larger than that among the non-astringent persimmon cultivars, and the average weight of individual fruits of the astringent persimmon cultivars was heavier than that of the non-astringent persimmon cultivars. The average TSS content of the astringent persimmon cultivars was lower than that of the non-astringent persimmon, however, the trend of the titratable acid was reversed. The huge differences of quality indexes among different cultivars might result from the different genetic origins.

During the edible period of persimmons, the difference of the peel color between the astringent persimmons and the non-astringent persimmons was similar to the difference that existed between red-fleshed and white-fleshed loquat fruits [18]. L* values and C* values of the astringent cultivars were slightly lower than those of the non-astringent cultivars, which reflected that the deeper color might be caused by the higher abundance of carotenoids. Through comparisons, the a* value of the astringent cultivars was higher, while the b* value was slightly lower, and as a result, the a*/b* ratio was higher. The a*/b* ratio was negative for green fruits, zero for yellow fruits, and positive for orange fruits [19]. Higher value of the positive ratio a*/b* indicated that the color was redder, therefore, the peel color of the astringent persimmons was redder than that of the non-astringent cultivars. Alternatively, the color could be well described by hue angle (*H*°) as follows: 0° for red-purple, 90° for yellow, and 180° for bluish-green and 270° for blue [20]. Therefore, the average hue angle of the astringent persimmon cultivars and non-astringent persimmon cultivars were 60° and 80°, respectively, indicating that the colors of these two kinds of persimmons tended to be orange and yellow, respectively.

**Table 1:** Major quality and color indexes in the fleshes of different persimmon cultivars.

Cultivars	W *^a^* (g)	VD *^b^* (cm)	HD *^c^* (cm)	FSI *^d^*	TSS *^e^* (ºBrix)	TA *^f^* (%)	*L^＊^*	*a^＊^*	*b^＊^*	*Hº*	C*	*a^＊^*^/^ *b^＊^*
*Astringent persimmons*												
Boaidashuishi	53.29	3.34	4.94	0.68	13.75	0.22	53.16	21.07	39.78	62.09	45.02	0.53
Changanhuoguan	70.82	4.58	4.96	0.92	14.78	0.38	45.28	20.14	28.48	54.73	34.88	0.71
Fujianding	136.73	5.54	6.26	0.88	15.46	0.25	41.16	14.88	23.15	57.27	27.52	0.64
Heixinshi	94.73	5.05	5.61	0.90	11.11	0.37	50.83	17.10	35.54	64.31	39.44	0.48
Heshi	339.35	6.63	9.38	0.71	16.25	0.19	56.04	25.68	44.60	60.07	51.46	0.58
Hiratanenashi	113.94	4.18	6.29	0.66	15.64	0.19	44.66	20.21	32.52	58.14	38.29	0.62
Hiro	101.73	4.82	5.92	0.81	15.20	0.18	43.67	5.17	25.14	78.38	25.67	0.21
Jianshi	122.70	6.33	5.69	1.11	16.31	0.30	48.06	15.49	32.93	64.81	36.39	0.47
Jinchengxiaoshi	66.27	3.67	5.22	0.70	16.80	0.18	47.66	24.35	34.17	54.53	41.96	0.71
Jinshi	277.98	7.56	7.45	1.01	13.24	0.20	48.11	15.90	32.06	63.62	35.79	0.50
Kangding No.1	77.08	4.28	5.08	0.84	18.57	0.20	52.02	25.04	40.37	58.19	47.51	0.62
Lantianshuishi	148.58	5.69	6.48	0.88	14.26	0.26	53.81	9.25	37.09	76.00	38.23	0.25
Mantianhong	209.03	5.92	7.72	0.77	16.57	0.17	42.96	29.32	29.58	45.25	41.65	0.99
Mendunshi	81.66	5.05	5.45	0.93	18.15	0.12	52.71	23.85	41.00	59.81	47.43	0.58
Miandanshi	87.20	4.92	5.37	0.92	17.60	0.33	47.23	13.94	31.14	65.88	34.12	0.45
Mimiguan	48.28	4.41	4.33	1.02	15.48	0.38	46.52	26.14	30.47	49.37	40.15	0.86
Naiyoushi	141.28	5.17	6.47	0.80	13.45	0.24	49.55	19.49	31.69	58.41	37.20	0.62
Ribenhongshi	96.64	4.29	5.82	0.74	18.57	0.17	47.77	23.43	32.07	53.85	39.72	0.73
Rongxianjingshi	183.40	5.60	6.68	0.84	21.15	0.25	39.79	22.39	24.16	47.18	32.94	0.93
Shagu No.1	106.00	4.72	5.97	0.79	15.01	0.23	43.54	13.36	26.61	63.34	29.78	0.50
Tianfushi	123.34	4.96	6.22	0.80	14.40	0.14	47.13	19.29	32.65	59.42	37.92	0.59
Tonewase	215.10	5.57	7.95	0.70	17.50	0.20	38.65	21.58	22.98	46.80	31.52	0.94
Xiaodishi	96.13	4.09	5.84	0.70	15.12	0.22	51.03	26.79	36.85	53.98	45.56	0.73
Xiaoercao	35.72	3.19	4.30	0.74	12.38	0.24	52.07	17.00	36.40	64.97	40.17	0.47
Xiaofangshi	138.00	4.83	6.49	0.74	14.53	0.33	47.53	17.01	31.80	61.86	36.06	0.53
Xinchangniuxinshi	97.78	6.14	5.15	1.19	13.04	0.33	51.04	19.73	37.49	62.24	42.36	0.53
Xingyangbaheshi	84.16	4.09	5.58	0.73	14.94	0.15	47.20	15.81	30.27	62.42	34.15	0.52
Yangshuohuoshi	63.60	3.71	5.20	0.71	14.25	0.16	47.18	13.92	34.92	68.27	37.59	0.40
Yichuanling	63.28	4.42	4.72	0.94	15.20	0.14	50.62	23.37	40.19	59.82	46.49	0.58
Yueshi	153.82	4.69	5.79	0.81	14.90	0.27	45.97	9.78	31.53	72.77	33.01	0.31
Zhaotianhong	159.40	4.86	7.21	0.67	15.50	0.20	41.31	21.67	21.02	44.13	30.19	1.03
Zhengyangjiandingshi	99.23	4.29	6.09	0.70	11.88	0.16	55.58	22.68	45.23	63.37	50.60	0.50
Average	121.45	4.89	5.99	0.82	15.34	0.23	47.81	19.21	32.93	59.85	38.46	0.60
*Non-astringent persimmons*												
Eshi No.1	143.65	4.90	7.06	0.69	15.38	0.20	43.74	-7.25	25.12	106.10	26.15	-0.29
Hanagosho	111.90	5.16	6.39	0.81	21.58	0.09	51.76	5.80	37.50	81.21	37.95	0.15
Jirou	114.54	4.41	6.64	0.66	13.34	0.11	64.00	3.49	50.55	86.05	50.67	0.07
Luotiantianshi	57.02	4.01	4.78	0.84	15.65	0.14	51.74	14.15	39.75	70.41	42.19	0.36
Matsumotowase	59.20	4.21	5.21	0.81	17.19	0.15	62.08	-11.14	47.66	103.16	48.94	-0.23
Nishimurawase	115.58	4.57	6.75	0.68	16.39	0.21	57.32	9.58	44.24	77.78	45.27	0.22
Okugosho	100.68	4.56	6.16	0.74	18.16	0.27	56.22	23.49	44.59	62.22	50.40	0.53
Sifangtianshi	24.44	2.82	3.51	0.80	19.77	0.14	49.29	10.80	34.10	72.43	35.77	0.32
Suruga	93.38	4.75	6.08	0.78	13.19	0.09	61.14	1.21	49.23	88.59	49.24	0.02
Uenishiwase	128.90	5.31	6.73	0.79	16.97	0.13	55.15	8.80	39.65	77.49	40.61	0.22
Xiangxitianshi	110.40	4.56	6.49	0.70	15.33	0.14	59.96	11.14	45.52	76.25	46.86	0.24
Xiaoguotianshi	28.29	3.32	3.83	0.87	24.76	0.12	55.30	8.53	44.68	79.19	45.49	0.19
Youhou	152.76	5.05	7.26	0.70	14.11	0.11	62.16	1.74	49.65	87.99	49.68	0.04
Zenjimaru	135.65	5.29	6.43	0.82	14.10	0.21	47.86	22.53	28.74	51.91	36.52	0.78
Average	98.31	4.49	5.95	0.76	16.85	0.15	55.55	7.35	41.50	80.06	43.27	0.19

*^a^* W: Weight; *^b^* VD: Vertical diameter; *^c ^*HD: Horizontal diameter; *^d^* FSI: Fruit shape index, The ratio of VD/HD; *^e^* TSS: Total soluble solid; *^f^* TA: Titrable acid.

### 2.2. HPLC chromatogram of carotenoids in persimmon fruit

**Figure 1:**
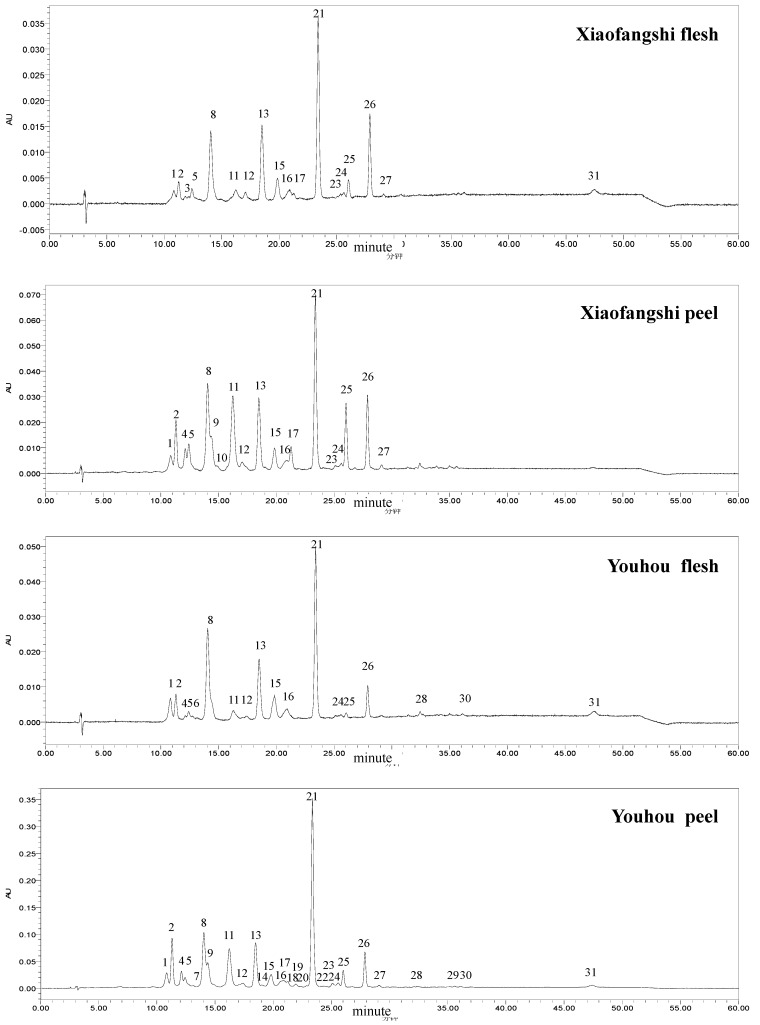
HPLC chromatograms of the extraction of the saponified carotenoids in the peel and the flesh of persimmon cv. ‘Xiaofangshi’ and ‘Youhou’, monitored at 450 nm. The peaks were numbered according to the elution sequence, see Table 2 for detail.

Thirty one specific carotenoids were detected in two persimmon cultivars, among which nine compounds were identified, including neoxanthin, violaxanthin, 9-*cis*-violaxanthin, lutein, zeaxanthin, β-cryptoxanthin, α-carotene, β-carotene and lycopene. On the other hand luteoxanthin, ζ-carotene and colorless carotenoids such as phytofluene, phytoene were not detected (Table 2). β-Cryptoxanthin was the most abundant carotenoids among all components in the peel and the flesh of ‘Xiaofanshi’ (astringent persimmon) and ‘Youhou’ (non-astringent persimmon), accounting for about 20-30% of the total carotenoids in both cultivars, which was consistent to the previous results [3,5]. In the peels of two cultivars, the contents of lutein were higher than those of zeaxanthin, while the contents of β-carotene were lower than those of lutein and zeaxanthin. The total contents of β-cryptoxanthin, lutein, zeaxanthin and β-carotene in ‘Xiaofangshi’ and ‘Youhou’ accounted for 49.09% and 51.48% of the total carotenoids, respectively. In the fleshes, the contents of zeaxanthin in both two cultivars were higher than those of β-carotene, while the contents of lutein were lower than those of β-carotene and zeaxanthin. The total contents of β-cryptoxanthin, zeaxanthin, β-carotene and lutein in ‘Xiaofangshi’ and ‘Youhou’ accounted for 59.47% and 49.67% of the total carotenoids, respectively. Among the nine specific carotenoids identified, 9-*cis*-violaxanthin was only detected in the peels of two cultivars, and was not detected in the flesh of both cultivars. The abundances of the carotenoid in peak 8 in two tissues of ‘Youhou’ were both the second, while those in the flesh and the peel of ‘Xiaofangshi’ were the second and third (Table 2), respectively. It was not identified in this study, but is worthy of further analysis and investigation. It could be seen in the HPLC chromatograms that the components of carotenoids in the peels and fleshes of both cultivars were similar (Figure 1). Although zeaxanthin cannot be converted into vitamin A in the human body, its protective effects on the neural cell of retina is well known [21]. There are few fruits that accumulate zeaxanthin, wolfberry fruit being a rich source for this substance [22]. 

**Table 2:** Separation and identification of carotenoid components in different tissues of the persimmon fruits cv. ‘Xiaofangshi’ and ‘Youhou’ by high performance liquid chromatography with photodiode array detection.

Peak no.	Carotenoids	Rt *^b^* (min)	λ_max_ *^c^* (nm)	Abundance *^d^* (%)			
				Xiaofangshi		Youhou	
				Peel	Flesh	Peel	Flesh
1	Neoxanthin	10.83	416, 440, 469	2.69	2.64	3.04	4.79
2	Violaxanthin	11.26	(415), 439, 468	5.73	3.87	7.14	4.18
3	Unidentified *^a^*	11.85	377, 423, 448	/ *^ e^*	0.65	/	/
4	Unidentified	12.13	412, 437, 463	2.57	/	2.84	0.51
5	Unidentified	12.40	423, 451, 470	3.51	1.62	2.23	1.46
6	Unidentified	12.71	401, 425, 451	/	/	/	0.47
7	Unidentified	13.12	429, 445, 469	/	/	0.45	/
8	Unidentified	14.05	417, 446, 474	13.11	15.07	10.98	23.72
9	9- *Cis*-violaxanthin	14.35	412, 435, 464	4.10	/	5.35	/
10	Unidentified	14.83	414, 442, 470	0.64	/	/	/
11	Lutein	16.22	(420), 445, 473	13.53	3.64	9.91	2.64
12	Unidentified	17.09	429, 457, 485	1.86	1.03	1.65	0.46
13	Zeaxanthin	18.51	(427), 450, 478	9.40	14.70	8.59	12.79
14	Unidentified	19.02	(425), 441, 475	/	/	0.49	/
15	Unidentified	19.86	(425), 441, 470	3.35	4.76	3.55	6.66
16	Unidentified	20.92	(427), 451, 474	2.11	3.19	2.87	4.12
17	Unidentified	21.27	419, 443, 477	2.93	1.06	1.10	/
18	Unidentified	21.94	422, 446, 481	/	/	0.72	/
19	Unidentified	22.40	(427), 454, 481	/	/	0.14	/
20	Unidentified	22.87	419, 443, 468	/	/	0.25	/
21	β-Cryptoxanthin	23.40	(426), 451, 479	19.34	29.58	28.55	29.44
22	Unidentified	24.35	429, 452, 479	/	/	0.34	/
23	Unidentified	25.09	419, 446, 476	0.52	0.65	0.87	/
24	Unidentified	25.63	(425), 451, 475	0.81	1.07	0.83	0.74
25	α-Carotene	26.04	(424), 446, 474	6.59	2.86	2.33	0.70
26	β-Carotene	27.90	(427), 452, 479	6.82	11.55	4.43	4.62
27	Unidentified	29.06	(425), 449, 474	0.40	0.64	0.30	/
28	Unidentified	32.44	425, 458, 485	/	/	0.14	0.69
29	Uunidentified	35.60	413, 436, 479	/	/	0.14	/
30	Unidentified	36.11	421, 451, 475	/	/	0.09	0.54
31	Lycopene	47.44	446, 473, 503	/	1.46	0.59	1.48

*^a^* Unidentified compounds having carotenoid spectra; *^b^* Rt = retention time; *^c^* Obtained with photodiode array detection in mobile solvents; *^d^* Expressed as percentage of total carotenoids; *^e^* Under the detection limit.

The results of this study indicated that a great amount of zeaxanthin could also be supplied by the persimmon fruit. Lycopene is the main pigment in tomato [23], watermelon [24], passionflower [25], and the red mutants such as orange, grapefruit and pomelo [26]. Lycopene was detected in the fleshes of both persimmon cultivars and in the peel of “Youhou”, however, it was not the main carotenoid in the persimmon fruit.

### 2.3. Contents of carotenoids in the flesh of different persimmon cultivars

The flesh is the edible part of the persimmon fruit, therefore, the contents of carotenoids in the flesh of the persimmon fruits of different cultivars were determined, and their RE values were also evaluated. The contents of the total carotenoids and eight specific carotenoids in the fleshes of different persimmon cultivars were significantly different, and the total content of carotenoids were between 194.61 ± 15.94 µg/100 g FW and 1,566.30 ± 116.33 µg/100 g FW (Table 3). Among eight specific carotenoids determined, β-cryptoxanthin and zeaxanthin were two specific carotenoids whose contents were the highest in all persimmon fleshes. These two components mentioned above accounted for 37.84-85.11% of the total carotenoids content. The content of β-cryptoxanthin in the flesh was between 60.91 ± 0.28 µg/100 g FW and 940.61 ± 77.50 µg/100 g FW, accounting for 21.33-63.92% of the total content of carotenoids. It was found that the average content of β-cryptoxanthin (381.01 µg/100 g FW) and its proportion in total carotenoids (47.40%) of the astringent persimmon were higher than the average content of β-cryptoxanthin (167.43 µg/100g FW) and its proportion in total carotenoids (37.58%) of the non-astringent persimmon by comparing the differences between the astringent persimmon and the non-astringent persimmon. The content of zeaxanthin in the flesh was between 5.57 ± 2.19 µg/100 g FW and 501.86 ± 38.25 µg/100 g FW, accounting for 10.53-32.14% of the total carotenoids content. It was found that the average content of zeaxanthin (162.15 µg/100 g FW) and its proportion in total carotenoids (19.04%) of the astringent persimmon were higher than the average content of zeaxanthin (70.16 µg/ 100 g FW) and its proportion in total carotenoids (17.12%) of the non-astringent persimmon by comparing the differences between the astringent persimmon and the non-astringent persimmon. In some cultivars, the contents of lutein, α-carotene and lycopene were under the detection limits. The difference of lycopene between different cultivars was larger than that of neoxanthin, violaxanthin, lutein and α-carotene. The contents of lycopene in the astringent cultivars ‘Mantanhong’ and ‘Tianfushi’, which were 112.67 ± 4.82 µg/100 g FW and 96.59 ± 2.34 µg/100 g FW, respectively, were relatively higher.

**Table 3:** The contents of carotenoids in the fleshes of different persimmon cutivars (μg/100 g FW).

Cultivars	T-car *^a^*	Neo *^b^*	Viola *^c^*	Lut *^d^*	Zea *^e^*	β-Crypto *^f^*	α-Car *^g^*	β-Car *^h^*	Lyc *^i^*	RE *^j^*
*Astringent persimmons*										
Boaidashuishi	1213.78	65.47	69.39	34.49	240.25	358.22	2.89	46.36	/ *^k^*	37.82
Changanhuoguan	519.37	22.16	13.82	21.89	108.46	222.56	4.31	16.51	/	21.66
Fujianding	602.23	21.88	22.25	7.58	71.84	334.27	3.02	36.54	/	34.20
Heixinshi	1292.42	48.28	47.43	11.09	166.35	678.38	3.32	48.54	/	64.90
Heshi	927.51	29.96	64.47	/	128.77	322.83	4.63	46.99	2.56	35.12
Hiratanenashi	373.18	18.37	16.68	14.25	63.80	169.86	2.39	12.86	3.99	16.50
Hiro	978.44	49.68	43.81	4.28	169.15	491.94	3.35	54.47	/	50.35
Jianshi	1085.88	22.36	21.63	30.59	208.46	555.19	5.88	39.45	8.95	53.33
Jinchengxiaoshi	458.74	13.39	12.89	/	57.96	271.11	3.33	25.33	6.15	27.09
Jinshi	352.91	5.68	4.52	2.74	86.42	192.78	2.25	25.14	/	20.44
Kangding No.1	931.33	11.98	20.08	9.75	157.55	486.79	6.25	83.92	/	55.07
Lantianshuishi	372.24	15.56	15.97	2.62	55.61	210.34	/	14.38	/	19.93
Mantianhong	1561.72	35.95	36.37	53.93	501.86	659.18	11.65	115.43	112.67	75.14
Mendunshi	450.52	13.12	12.24	8.25	75.43	261.88	4.64	19.52	/	25.46
Miandanshi	680.40	29.15	22.20	13.11	186.28	237.65	6.70	37.84	/	26.67
Mimiguan	462.05	15.66	11.60	17.19	93.08	183.72	3.53	18.68	26.43	18.72
Naiyoushi	338.90	16.82	9.06	5.66	68.55	158.35	6.78	34.68	7.10	19.54
Ribenhongshi	297.45	16.79	7.58	8.04	70.80	104.42	/	18.75	13.02	11.83
Rongxianjingshi	1566.30	26.73	18.32	19.91	433.91	793.35	7.62	69.14	25.33	78.27
Shagu No.1	958.74	20.73	24.74	29.96	203.54	447.25	5.63	36.61	29.90	43.84
Tianfushi	777.28	36.63	19.79	12.94	136.40	268.41	5.20	64.04	96.59	33.47
Tonewase	580.73	40.63	48.37	5.83	65.68	191.22	2.22	22.06	29.47	19.80
Xiaodishi	383.52	8.75	15.67	2.85	53.02	210.32	/	19.81	/	20.83
Xiaoercao	1562.73	24.70	54.39	55.10	364.65	757.87	7.67	65.00	11.30	74.63
Xiaofangshi	335.63	16.73	14.31	11.13	53.64	123.96	9.53	39.44	5.06	17.70
Xinchangniuxinshi	1471.43	23.95	15.31	33.32	311.70	940.61	8.54	68.83	15.99	90.57
Xingyangbaheshi	1066.04	15.86	18.16	7.76	264.39	520.71	5.94	67.31	12.66	55.11
Yangshuohuoshi	930.45	35.00	27.16	19.70	187.15	521.05	3.92	27.98	/	48.41
Yichuanling	1441.48	75.11	94.34	32.82	235.84	443.51	4.09	61.46	/	47.54
Yueshi	492.57	22.46	16.77	/	86.95	281.20	/	16.09	4.05	26.12
Zhaotianhong	1274.28	35.59	48.80	27.35	134.23	599.18	9.50	91.07	41.9	65.90
Zhengyangjiandingshi	771.27	14.14	15.21	3.69	146.99	418.12	2.55	28.30	/	39.77
Average	821.72	26.54	27.60	15.87	162.15	388.01	4.60	42.89	14.62	39.87
*Non-astringent persimmons*										
Eshi No.1	285.51	18.77	15.69	25.28	47.14	60.91	5.45	25.88	/	9.84
Hanagosho	325.14	11.29	10.01	16.04	61.99	118.96	5.96	32.90	/	15.89
Jirou	624.42	32.07	19.21	19.90	49.27	208.05	5.14	38.47	33.53	24.18
Luotiantianshi	439.07	30.55	15.70	25.79	85.29	182.49	5.41	19.13	/	18.85
Matsumotowase	519.44	24.30	12.75	19.62	110.79	189.41	9.05	49.76	26.86	24.83
Nishimurawase	391.81	14.32	10.07	20.66	75.50	203.19	5.60	41.39	6.86	24.76
Okugosho	350.70	16.65	8.90	20.51	67.67	141.97	5.17	20.45	17.49	15.67
Sifangtianshi	567.59	20.10	16.81	9.36	74.65	274.54	2.50	22.84	36.56	26.89
Suruga	284.65	11.90	9.77	11.57	78.01	98.11	2.82	15.16	2.91	10.94
Uenishiwase	194.61	10.31	4.60	13.44	52.92	71.23	2.97	14.63	/	8.62
Xiangxitianshi	339.19	29.02	11.44	18.50	35.57	144.38	7.89	24.54	7.20	16.78
Xiaoguotianshi	728.84	29.59	19.93	27.55	91.27	304.38	14.19	53.13	/	35.40
Youhou	500.13	43.31	24.84	13.760	66.67	175.59	4.24	24.99	8.37	19.15
Zenjimaru	664.98	31.99	38.92	19.73	85.50	170.82	9.15	46.85	56.60	22.81
Average	444.01	23.16	15.62	18.69	70.16	167.43	6.11	30.72	14.03	19.58

*^a^* Total carotenoids; *^b^* Neoxanthin; *^c^* Violaxanthin; *^d^* Lutein; *^e^* Zeaxanthin; *^f^* β-Cryptoxanthin; *^g^* α-Carotene; *^h^* β-Carotene;*^ i^* Lycopene; *^j^* Retinol equivalent; *^k^* Under the detection limit.

β-Cryptoxanthin, α-carotene and β-carotene can be converted into vitamin A in animals and humans. Vitamin A value in fruit can be expressed by μg retinol equivalent (μg RE), and 1 μg RE equals to 6 μg β-carotene or 12 μg other carotenoids of vitamin A resource, such as β-cryptoxanthin or α-carotene [7]. In all the cultivars tested, the RE value was the highest in ‘Xinchangniuxinshi’ (90.57 µg/100 g FW); while it was the lowest in ‘Uenishiwase’(8.62 µg/100 g FW). The average RE values of the astringent persimmons and the non-astringent persimmons were 39.87 µg/100 g FW and 19.58 µg/100 g FW, respectively. 

### 2.4. The contents of carotenoids in persimmon fleshes in different stages of maturation

The contents of carotenoids in the flesh of ‘Yueshi’ fruit rapidly increased from the green mature period to the soft mature period, and except for lutein and lycopene, the trends of other specific carotenoids were similar to that of the total carotenoids. The content of lutein decreased during fruit maturation, and could not be detected in the soft mature period, while lycopene only existed in a small amount in the persimmon in soft mature period, while α-carotene was not detected in the three different stages of maturation. In the green mature period, the content of total carotenoids was only 83.26 ± 0.50 μg/100 g FW. With the maturation of the fruit, the contents of the total carotenoids in the half mature period and soft mature period were 284.39 ± 10.96 μg/100 g FW and 492.57 ± 25.03 μg/100 g FW, respectively (Table 5), which were 3.42 and 5.92 times of that in green mature period. The total amounts of β-cryptoxanthin and zeaxanthin in the three mature periods mentioned above accounted for 36.91%, 51.34% and 74.74% of the total carotenoids, respectively. It meant that β-carotene, β-cryptoxanthin and zeaxanthin were mainly produced in the cyclic branch of lycopene [27], and with the maturation of the fruit, the increase of the gene expression level of β-carotene hydroxylase (*HYb*) led to more conversion of β-carotene to β-cryptoxanthin and zeaxanthin [28].

**Table 4:** The contents of carotenoids in the fleshes of persimmon fruit cv. ‘Yueshi’ in different stages of maturation (μg/100 g FW).

Mature stage	T-car *^a^*	Neo *^b^*	Viola *^c^*	Lut *^d^*	Zea *^e^*	β-Crypto *^f^*	α-Car *^g^*	β-Car *^h^*	Lyc *^i^*	RE *^j^*
Green mature	82.36	9.86	5.93	7.98	14.24	16.16	5.24	/	2.22	2.22
Half ripening	284.39	17.07	13.01	5.57	40.60	105.41	10.97	/	10.61	10.61
Soften ripening	492.57	22.46	16.77	/ *^ k^*	86.95	281.20	16.09	4.05	26.12	26.12

*^a^* Total carotenoids; *^b^* Neoxanthin; *^c^* Violaxanthin; *^d^* Lutein; *^e^* Zeaxanthin; *^f^* β-Cryptoxanthin; *^g^* α-Carotene; *^h^* β-Carotene;*^ i^* Lycopene; *^j^* Retinol equivalent; *^k^* Under the detection limit.

## 3. Experimental

### 3.1. Plant materials and sampling

In this study, the fruits of 46 persimmon (*Diospyros kaki* L.) cultivars were used, including 32 astringent and 14 non-astringent ones (Table 1). During the edible period of the persimmons, the fruits of different persimmon cultivars were collected from the National Persimmon Germplasm Nursery in Northwest A&F University (Yangling, Shanxi, China), Zhenjiang Agricultural Science Institute (Jurong, Jiangsu, China) and Persimmon Germplasm Experimental Station of Huazhong Agricultural University (Wuhan, Hubei, China) and then transferred to the lab. Ten intact fruits in each cultivar were selected to determine the main quality indexes, including the fruit size, shape, color, TSS (Total Soluble Solid) content and titratable acid content, and the results were listed in Table 1. After that, the pedicle was removed, and the persimmon fruit was divided into edible part (flesh) and inedible part (peel), and then they were cut into small pieces and frozen in liquid nitrogen, and then stored at -20 °C for analysis.

### 3.2. Fruit quality analysis

The fruit weight was determined by balance. The vertical and horizontal diameter of the fruit was measured by a vernier caliper. The fruit shape index is the ratio of the vertical and horizontal diameters. TSS was determined by a portable refractometer (Chengdu Optical Instrument Factory, China), and the measurements were performed on the opposite sides of the equatorial plane on each fruit. All the indexes mentioned above were the mean value of 10 fruits. Titratable acid was determined according to the titration method as follows: flesh from 10 fruits (1 g) was ground with distilled water (5 mL). After filtration and centrifugation for 10 min at 10,000 g, the supernatant was brought to 10 mL with distilled water. The water was heated for 5 min at 100 °C to eliminate CO_2_, and subsequently titrated with fresh 10 mM NaOH to pH 8.2. Each sample was repeated for three times.

### 3.3. Determination of color

The color of the opposite sides of the equatorial plane on each fruit were determined by a TC-P2A automatic reflectance spectrophotometer (Beijing Xinaoyike Photoelectric Technology Co. Ltd., China) using three color parameters including L*, a* and b* values. Hue angle (*H*°= arctangent (b*/a*)) and chroma (C* = (a*^2^ + b*^2^)^1/2^) were calculated by the method reported before [29]. Ten fruits were continuously determined to calculate the mean value.

### 3.4. Extraction and analysis of carotenoids

Extraction of carotenoids was performed according to the method reported by Zhou *et al*. with some modifications [18]. A suitable amount of the peel or the flesh was collected, liquid nitrogen was added, and then they were fully ground. Subsequently, sample (200 mg) was weighed and transferred to a 2 mL centrifuge tube, and methanol (350 μL) was added in and mixed homogeneously; then chloroform (700 μL) was added in and mixed homogeneously; then 10% NaCl solution (350 μL) was added and the mixture was centrifuged at 15 °C and 8,000 g for 5 min, and then the chloroform phase was collected. Precipitates were extracted with chloroform (350 μL) and the process was repeated for several times till they became colorless. Then the chloroform phases were combined and dried under N_2_. The extract was dissolved in diethyl ether (50 μL), and then 6% methanolic KOH solution (350 μL) was added in and incubated in the dark at 60 °C for 30 min. Then chloroform (700 μL) and 10% NaCl solution (350 μL) were added and mixed homogeneously, and centrifuged at 15 °C and 8,000 g for 5 min. Then the chloroform phase was collected. 10% NaCl solution (700 μL) was added to the chloroform phase to extract KOH and this step was repeated several times till the water phase became neutral. The chloroform phase was blown to dryness using N_2_, and then stored at -20 °C for HPLC analysis. In order to prevent photodegradation, isomerization and structure variation, the extraction of carotenoids was performed in dim light. The extraction of each sample was repeated three times.

Before HPLC analysis, the dried extract was dissolved in ethyl acetate (HPLC grade, 120 μL) and then centrifuged, and the supernatant was used for HPLC analysis. Carotenoids were analyzed on a Waters HPLC-PDAD system (Waters Corp., Milford, MA), using a 5 μm C_30_ reverse phase column (250 × 4.6 mm) and 20 × 4.6 mm C_30_ guard-column and an external standard method. The column temperature was 25 °C, and the elution conditions followed the method reported by Xu *et al.* [26]. The results were analyzed by Waters Empower software, and carotenoids were identified by both the retention times and absorption spectrum curves. Lutein, zeaxanthin, β-cryptoxanthin, α-carotene and β-carotene standards were all purchased from Sigma Chemical Co. (St. Louis, MO, USA). Neoxanthin, violaxanthin, luteoxanthin, 9-*cis*-violaxanthin, phytofluene, phytoene, ζ-carotene and lycopene were prepared according to previous methods [26,30]. 

## 4. Conclusions

In the present study, chromatograms of carotenoids in persimmon fruit were recorded. Nine specific carotenoids (*i.e.,* neoxanthin, violaxanthin, 9-*cis*-violaxanthin, lutein, zeaxanthin, β-cryptoxanthin, α-carotene, β-carotene and lycopene) were identified. Although the contents of the total carotenoids in the fleshes were great different from each other among different persimmon cultivars, β-cryptoxanthin and zeaxanthin were two specific carotenoids whose contents were the highest among all the persimmon flesh samples. Therefore, persimmon flesh could be utilized as one of the few abundant resources of zeaxanthin besides as a vitamin A source food. Besides the cultivar, the maturity period was also an important factor influencing the contents of carotenoids and RE values in the persimmon flesh samples.
